# Inter-platform agreement and diagnostic performance of the automated LIAISON^®^ chemiluminescence immunoassay vs. the Sofia^®^ fluorescence immunoassay for *Streptococcus pneumoniae* and *Legionella pneumophila* urinary antigen detection

**DOI:** 10.3389/fmicb.2026.1926157

**Published:** 2026-09-21

**Authors:** Pablo Pillado, Alba Llop, Tessa Katherina Vela, Asunción Nathaly Ladines, Montserrat Giménez, Pere-Joan Cardona, Adrián Antuori

**Affiliations:** 1 Microbiology Department, North Metropolitan Clinical Laboratory, Germans Trias i Pujol University Hospital, Badalona, Spain; 2 Genetics and Microbiology Department, Universitat Autònoma de Barcelona, Cerdanyola del Vallès, Spain; 3 Centro de Investigación Biomédica en Red de Enfermedades Respiratorias (CIBERES), Madrid, Spain

**Keywords:** chemiluminescence immunoassay, community-acquired pneumonia, fluorescence immunoassay, *Legionella pneumophila*, *Streptococcus pneumoniae*, urinary antigen test

## Abstract

**Background:**

Urinary antigen tests allow rapid diagnosis of community-acquired pneumonia (CAP) caused by *Streptococcus pneumoniae* and *Legionella pneumophila*. We compared the automated LIAISON^®^ chemiluminescence immunoassay (CLIA) with the Sofia^®^ fluorescence immunoassay (FIA).

**Methods:**

This retrospective, single-center study analyzed residual urine from adults with suspected CAP (December 2024–February 2026). The primary analysis compared initial results across both platforms. Our primary endpoint evaluated Cohen's kappa alongside overall, positive, and negative percent agreement (OPA, PPA, NPA). Restricted exclusively to *S. pneumoniae*, the secondary endpoint assessed diagnostic accuracy against a composite reference standard comprising blood culture, sputum culture, and respiratory PCR. Furthermore, we executed two sensitivity analyses. One incorporated a resolution protocol for initially discordant results. Another restricted the reference standard strictly to culture-confirmed cases. Finally, a Stability cohort (storage ≤ 60 days) was analyzed alongside the Total cohort.

**Results:**

We analyzed 318 patients in the *S. pneumoniae* arm and 335 in the *L. pneumophila* arm. For *S. pneumoniae*, agreement was substantial (OPA 85.2%, PPA 57.7%, NPA 98.6%, kappa 0.63) and the LIAISON^®^ assay was less often positive (19.8% vs. 32.7%, *p* < 0.001). Apparent sensitivity favored the Sofia^®^ FIA in every comparison, significantly in the primary analysis (Total 77.1% vs. 62.5%, *p* = 0.039; Stability 86.8% vs. 71.1%, *p* = 0.031). Neither the secondary analysis nor the *post-hoc* culture-restricted reference standard altered the direction of any comparison. Under this strict standard, statistical significance persisted exclusively within the Stability cohort prior to discrepancy resolution. For *L. pneumophila*, agreement was substantial (OPA 93.4%, PPA 66.7%, NPA 98.2%, kappa 0.72), with the LIAISON^®^ assay again less often positive (11.6% *vs*. 15.2%, *p* = 0.017).

**Conclusion:**

Ultimately, both platforms demonstrated substantial diagnostic agreement across both pathogens. NPA consistently exceeded 98%. However, PPA remained strictly moderate (< 74%). Consequently, the LIAISON^®^ CLIA yielded fewer positive determinations overall. Regarding *S. pneumoniae*, this profile drove higher apparent specificity alongside lower apparent sensitivity against our composite reference standard. Crucially, these variances remain inseparable from inherent pre-analytical asymmetries between fresh and frozen-thawed specimens. Simultaneous prospective fresh-specimen clinical evaluations are strictly required.

## 1 Introduction

Community-acquired pneumonia (CAP) remains a leading cause of infection-related hospitalization and death worldwide (GBD 2023 Causes of Death Collaborators, 2025), and *Streptococcus pneumoniae* remains the most frequently identified pathogen, followed by other clinically relevant causes such as *Legionella pneumophila* (Torres et al., 2021).

Early identification of the causative pathogen in CAP is essential to guide appropriate antibiotic therapy, limit the emergence of antimicrobial resistance, and reduce drug-related toxicity (Kim et al., 2022). However, conventional methods, including blood and respiratory cultures, have low sensitivity and prolonged turnaround times (Blaschke, 2011; Yu et al., 2022).

Urinary antigen tests (UATs) are rapid, non-invasive, and easy-to-perform diagnostic tools that facilitate the early initiation of targeted antimicrobial therapy and improve clinical management (Murdoch et al., 2001). The ATS/IDSA guidelines restrict UATs primarily to severe cases or specific epidemiological risks (Metlay et al., 2019), whereas the Spanish SEPAR guidelines recommend universal testing for all hospitalized CAP patients due to a higher regional *Legionella* prevalence (Menéndez et al., 2020). For *S. pneumoniae*, the reported overall sensitivity ranges from 60% to 80%, with specificities generally above 90% (Rosón et al., 2004; Said et al., 2013). For *L. pneumophila* (mainly serogroup 1), sensitivity ranges from 70% to 90%, and specificity is usually greater than 95% (Shimada et al., 2009; Pierre et al., 2017).

Several automated platforms are currently available for the detection of urinary antigens. The Sofia^®^ system (Quidel Corporation, San Diego, CA, USA) combines lateral flow immunoassay technology with automated fluorescence reading, providing a more standardized and objective interpretation of results than conventional colorimetric lateral flow tests (Beraud et al., 2015; Euser et al., 2018). More recently, the LIAISON^®^ platform (DiaSorin, Saluggia, Italy) has introduced a fully automated chemiluminescence immunoassay (CLIA) that enables continuous, high-throughput processing (Ferraiuolo et al., 2025). The two *Legionella* assays differ in antigenic target: the Sofia^®^ FIA detects the lipopolysaccharide of *L. pneumophila* serogroup 1, whereas the LIAISON^®^ assay is a dual-antigen format that additionally detects the peptidoglycan-associated lipoprotein, conserved across *Legionella* serogroups and species (Ferraiuolo et al., 2025).

Three studies have reported clinical performance data for the LIAISON^®^ *Legionella* Urinary Ag assay, with reported agreement against enzyme immunoassay comparators ranging from 86.3% to 98.0% (Ferraiuolo et al., 2025; Cusato et al., 2026; Azzato et al., 2026). For *S. pneumoniae*, we are not aware of any published comparison of the LIAISON^®^ Ag assay with the Sofia^®^ FIA, and clinical performance data remain particularly scarce.

This study aimed to evaluate the diagnostic performance of the LIAISON^®^ platform through two pre-specified analyses. First, we assessed the inter-platform agreement between the LIAISON^®^ CLIA and the Sofia^®^ FIA for *S. pneumoniae* and *Legionella* urinary antigens. Second, we evaluated the accuracy against a composite reference standard.

## 2 Materials and methods

### 2.1 Study design and ethical considerations

A single-center, retrospective comparative diagnostic accuracy study was conducted at the Microbiology Department of the North Metropolitan Clinical Laboratory, Germans Trias i Pujol University Hospital (Badalona, Spain), between December 2024 and February 2026.

The study evaluated the fully automated LIAISON^®^ chemiluminescence immunoassay (CLIA; DiaSorin, Saluggia, Italy), comprising the LIAISON^®^ *S. pneumoniae* Ag and LIAISON^®^ *Legionella* Urinary Ag assays. This platform was compared with the semiautomated Sofia^®^ *S. pneumoniae* and *Legionella* fluorescence immunoassays (FIA; Quidel Corporation, San Diego, CA, USA) currently used in routine practice.

The study was conducted in accordance with the principles of the Declaration of Helsinki and was approved by the hospital's Research Ethics Committee (reference PI-24-250; 10 January 2025) and complied with all institutional regulations. Due to the study's retrospective nature, the committee waived the requirement for informed consent, and all data were analyzed anonymously.

### 2.2 Data collection

For each included patient, all microbiological data were retrieved from the laboratory information system. We also recorded the date of the initial routine Sofia^®^ analysis, the date of the comparative LIAISON^®^ analysis, and the availability of respiratory culture or PCR.

This retrospective study was conducted in three distinct stages by three independent teams. The specimen retrieval team selected the residual samples. This first team had full access to the laboratory information system. Consequently, they were aware of both the routine Sofia^®^ results and the reference-standard results. The analytical team performed the LIAISON^®^ determinations. This second team was strictly blinded to the reference-standard results. However, they were not blinded to the initial Sofia^®^ results. The pre-specified discrepancy-resolution protocol (Section 2.5) explicitly required a direct comparison between these two antigen platforms. Finally, a dedicated statistical team performed the data analysis. This third team did not conduct any laboratory testing. They were fully aware of the platform of origin for each result.

### 2.3 Patient selection and specimen collection

Patients were not enrolled consecutively. All patients were identified retrospectively from residual material held in storage. Eligibility criteria were applied at that point. Selection was performed at the specimen level. Within each arm, every specimen originated from a unique patient.

Patients were included if they met the following criteria: (i) age ≥18 years with clinical and radiological suspicion of CAP; (ii) prior urinary antigen testing via the routine laboratory platform; (iii) a residual urine volume of ≥1.0 mL to allow for comparative analysis and discrepancy resolution, and (iv) for the *S. pneumoniae* arm, availability of an adequate-quality respiratory culture (Murray–Washington grade ≥3) or isolation of *S. pneumoniae* from blood culture. Samples failing to meet these criteria were excluded.

For the *L. pneumophila* arm, an enriched design was applied to obtain an adequate number of target-positive cases, enrolling etiology-negative CAP patients together with confirmed *Legionella* cases. Candidate confirmed cases were identified based on a positive routine urinary antigen test (UAT), a positive respiratory culture, or a positive polymerase chain reaction (PCR) on a sputum specimen. Crucially, the routine UAT result was used solely for specimen selection and was deliberately excluded from the reference standard to avoid incorporation bias.

The study used two different composite reference standards. For *S. pneumoniae*, the eligibility criteria ensured that every enrolled patient provided a suitable specimen and received a clear classification. A patient was classified as positive if the microorganism was isolated in a blood culture, an adequate-quality sputum culture (Murray–Washington grade ≥3) (Murray and Washington, 1975), or detected through a respiratory PCR test from adequate-quality sputum. Although a specific quantitative threshold, such as cycle threshold (Ct) values, was not established to define positivity for *S. pneumoniae* by sputum PCR, a qualitative rule was applied. Molecular detection was accepted as a positive result for the composite reference standard only when *S. pneumoniae* was the sole pathogen detected by the molecular assay. Because respiratory specimens may carry *S. pneumoniae* as a colonizer, this criterion reduces but does not eliminate the possibility that a positive molecular result reflects carriage rather than the etiology of the pneumonia. Patients who met the eligibility criteria and in whom no positive result was obtained among the reference tests actually performed were classified as reference-standard negative. A negative classification therefore means that no positive result was obtained among the tests performed. It does not mean that all three reference tests were performed and were negative. Inclusion criterion (iv) ensured that no patient could be classified as reference-standard negative without at least one evaluable culture-based method, either an adequate-quality respiratory culture or a blood culture.

In contrast, the composite reference standard for *L. pneumophila* required a sputum specimen. Positivity was defined exclusively by isolation in a sputum culture or detection by PCR in a sputum specimen. Because both methods require a sputum sample, a reference classification could only be established for the subset of patients who provided this specimen. Consequently, patients without a sputum specimen were excluded from the *L. pneumophila* diagnostic accuracy analysis, though they were retained for the inter-platform agreement analysis. This represents a key structural difference from the *S. pneumoniae* arm, which is addressed in the Discussion.

### 2.4 Laboratory assays and procedures

All specimens had been initially tested as part of routine clinical practice using the Sofia^®^ *S. pneumoniae* FIA and Sofia^®^ *Legionella* FIA (Quidel, San Diego, CA, USA) on fresh urine samples. These assays use lateral flow immunofluorescence technology and provide objective readings through the Sofia^®^ analyzer. Following routine analysis, residual urine volumes were refrigerated (2–8 °C) and frozen at −20 °C within 72 hours of collection. Each specimen was thawed once, immediately before LIAISON^®^ testing. As described in Section 2.5, specimens requiring discrepancy resolution were re-tested from that same thawed aliquot without re-freezing.

To evaluate and mitigate potential storage-related antigen degradation, two analytical cohorts were established: the total cohort, which included all available specimens regardless of storage duration, and the Stability cohort, which was restricted to specimens stored for 60 days or less. Because the stability cohort is a subgroup of the Total cohort rather than an independent series, differences between the two cohorts are interpreted as exploratory.

For the comparative arm, frozen aliquots were thawed, centrifuged (3,000 × g for 10 min), and analyzed using the LIAISON^®^ *S. pneumoniae* Ag and LIAISON^®^ *Legionella* Urinary Ag assays (DiaSorin, Saluggia, Italy). These one-step CLIA assays employ magnetic microparticles coated with specific antibodies and were run on a single fully automated LIAISON^®^ XL analyzer (serial number 2210001820, software version 4.2.6.2), strictly following the manufacturer's protocols.

The LIAISON^®^ positivity cut-offs were ≥1.00 for *S. pneumoniae* and ≥0.70 for *L. pneumophila*. All determinations were processed under valid calibrations alongside required positive and negative controls. Numeric LIAISON^®^ indices are available for all determinations. Conversely, the Sofia^®^ analyzer did not retain historical numeric records and the laboratory information system stores only the final qualitative result. Numeric Sofia^®^ indices from initial determinations were retrievable for only 7 of the 318 pneumococcal and 5 of the 335 *Legionella* patients analyzed. Sofia^®^ indices were available for re-tested determinations following discrepancy resolution.

### 2.5 Discrepancy resolution

Discordant results between the two platforms were resolved using pathogen-specific protocols for a secondary analysis (Figure 1). For both pathogens, the rationale was pre-analytical. The routine Sofia^®^ result had been obtained on fresh urine, whereas the LIAISON^®^ was performed later on a frozen and thawed aliquot. Because freeze-thaw cycling and prolonged storage can alter urinary antigen detection (Wong et al., 2021a), an initial discordance could be caused by specimen handling rather than by a true difference between the two methods. Since the LIAISON^®^ assay had already been performed on the thawed aliquot, discrepancy resolution consisted of repeating Sofia^®^ FIA on that same aliquot, without any further freeze–thaw cycle.

**Figure 1:**
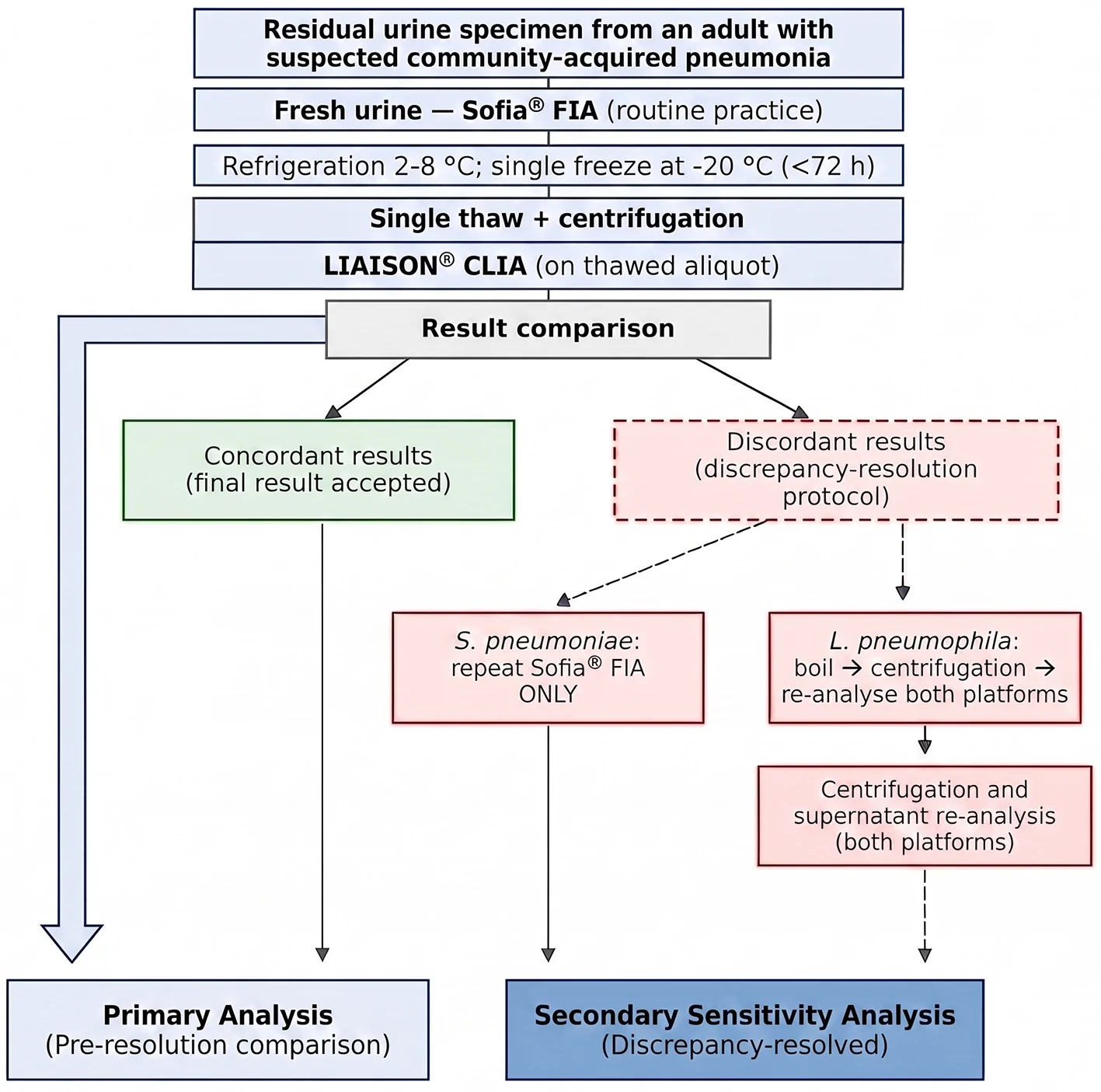
Analytical workflow and discrepancy resolution algorithm. The Sofia^®^ fluorescence immunoassay (FIA) result was obtained on fresh urine during routine clinical practice. Residual urine was subsequently frozen once, thawed, centrifuged, and analyzed with the LIAISON^®^ chemiluminescence immunoassay (CLIA). The initial comparison of these first determinations across both platforms constitutes the primary analysis. Concordant results directly determined the final status for all analyses. Discordant results were subjected to pathogen-specific resolution protocols without any further freeze–thaw cycles. For *S. pneumoniae*, immediate repeat testing with the Sofia^®^ FIA only. For *L. pneumophila*, boiling (100 °C for 5–10 min) followed by centrifugation and re-analysis of the supernatant on both platforms. The repeat results from these discordant specimens were exclusively utilized for the secondary sensitivity analysis (discrepancy-resolved)

For *S. pneumoniae*, the thawed aliquot was re-tested with the Sofia^®^ FIA, and the repeat result was considered definitive for analysis against the reference standard. The LIAISON^®^ assay was not repeated in this arm. The first LIAISON^®^ determination is therefore also the final one, and the two analyses differ only through the Sofia^®^ repeat.

For *L. pneumophila*, a heat-treatment protocol was used: urine samples were boiled at 100 °C for 5–10 min to precipitate thermolabile proteins and eliminate non-specific interference (Beraud et al., 2015). Following centrifugation, the supernatant was re-analyzed on both platforms to obtain the final result (Figure 1).

### 2.6 Sample size

The sample size was driven by the primary endpoint: the inter-platform agreement between the LIAISON^®^ CLIA and the Sofia^®^ FIA. According to CLSI EP12 guidelines [Clinical and Laboratory Standards Institute (CLSI), 2023], at least 60 positive and 60 negative specimens are sufficient to demonstrate a percent agreement with a lower 95% confidence interval (CI) bound of ≥90%. Based on budget constraints and kit availability, a pragmatic target of 300 specimens per assay was pre-specified. Compliance with the CLSI requirement of at least 60 positive and 60 negative specimens was verified stratum by stratum against the primary data. It was met for the comparator-negative stratum in both arms and for the comparator-positive stratum of the *S. pneumoniae* arm, but not for the comparator-positive stratum of the *L. pneumophila* arm. Positive percent agreement and Cohen's kappa for that arm therefore lack the precision intended by the CLSI target and are reported strictly as descriptive estimates.

Diagnostic sensitivity and specificity against the composite reference standard were secondary, descriptive endpoints. A 19% prevalence of confirmed pneumococcal pneumonia was assumed based on regional meta-analysis data (Navarro-Torné et al., 2021). Under this assumption, a target of 300 patients would yield roughly 57 culture- or PCR-confirmed cases, allowing sensitivity to be estimated with a 95% CI half-width of ±12%. For the *L. pneumophila* arm, the low number of expected cases precluded a reliable estimation of sensitivity and specificity; therefore, the analysis was restricted to inter-platform agreement. All accuracy estimates are reported with their 95% CIs in accordance with STARD 2015 guidelines (Bossuyt et al., 2015).

### 2.7 Statistical analysis

Two comparisons were analyzed for each pathogen and cohort. The primary analysis was the pre-resolution comparison of the first determination on each platform. The secondary analysis was the comparison of the final results obtained after the discrepancy-resolution protocol. Because only discordant patients entered the resolution protocol, that procedure can only move pairs from discordance to concordance, and it can therefore only raise the agreement estimates. The same first-vs.-first definition was applied to both arms.

Analyses were stratified by microorganism and conducted across both the previously defined Total and Stability cohorts. Statistical analysis was conducted using IBM SPSS Statistics, version 31.0 (IBM Corp., Armonk, NY, USA).

Inter-platform agreement for both microorganisms was evaluated following CLSI EP12 guidelines [Clinical and Laboratory Standards Institute (CLSI), 2023]. This agreement was determined using Cohen's kappa index (κ), interpreted according to Landis and Koch criteria (Landis and Koch, 1977). Additionally, overall, positive, and negative percent agreements (OPA, PPA, NPA) were calculated. All metrics include Wilson score 95% confidence intervals (CIs).

Diagnostic accuracy was reported following the STARD 2015 statement (Bossuyt et al., 2015). For the *S. pneumoniae* arm, we present a cross-tabulation against the reference standard and the derived accuracy estimates [sensitivity, specificity, positive predictive value (PPV), and negative predictive value (NPV)] with 95% CIs. Positive and negative likelihood ratios were calculated for this arm with 95% CIs using the log method.

Paired differences between assays were evaluated using the exact conditional binomial (McNemar) test. To ensure exact compatibility between hypothesis testing and interval estimation, interval estimates for paired comparisons were reported on the conditional scale. This scale represents the proportion of discordant pairs in the direction of the specified difference. These estimates were calculated using exact Clopper–Pearson 95% confidence intervals. Furthermore, the exact number of discordant pairs is explicitly reported for every paired comparison. Absolute differences between assays in percentage points were provided solely as descriptive point estimates. Confidence intervals for single proportions were calculated using the Wilson score method. Additionally, confidence intervals for Cohen's kappa were derived asymptotically. Finally, statistical significance was defined as a *p*-value of less than 0.05.

The sample size was calculated to estimate sensitivity and specificity with a pre-specified precision (Section 2.6). It was not calculated to provide statistical power for a paired comparison between assays. Furthermore, the study was not designed as a non-inferiority or equivalence trial. Consequently, a non-significant difference should not be interpreted as evidence of equivalence.

Diagnostic performance was assessed against the pre-specified composite reference standard as a whole, rather than against each reference method separately. Evaluating each method individually would yield unstable stratified estimates due to the small number of identified cases. A descriptive breakdown by reference method is nevertheless provided separately.

Conversely, due to the low number of reference-confirmed cases and the cohort's enrichment with positive cases, the *L. pneumophila* arm excludes these accuracy estimates and is restricted to inter-platform concordance metrics.

Additionally, we performed a single *post-hoc* restricted analysis for diagnostic accuracy of the pneumococcal arm (Section 3.2.5). This specific analysis applied a distinct rule to each stratum of the reference standard. A patient was considered reference-positive only if *S. pneumoniae* was successfully isolated from either a blood culture or an adequate-quality respiratory culture. Conversely, a patient was classified as reference-negative only if both of these cultures were performed and neither yielded the pathogen. Furthermore, cases classified as positive by respiratory PCR alone were strictly excluded rather than reclassified as reference-negative. This exclusion was necessary because their true diagnostic status could not be definitively established.

## 3 Results

### 3.1 Study samples and microbiological workup

The initial number of patients reviewed before applying eligibility criteria was not recorded. Similarly, the number of specimens rejected for a residual volume below 1.0 mL was not documented. A total of 663 paired inter-platform comparisons were performed on 594 distinct urine specimens from adult patients with clinical and radiological suspicion of CAP between December 2024 and February 2026. Of these, 653 paired comparisons, corresponding to 589 distinct patients, entered the analyses: 318 in the *S. pneumoniae arm* (328 processed, 10 excluded because inclusion criterion (iv) was not met) and 335 in the *L. pneumophila arm* (no exclusions). Sixty-four patients were tested in both arms (Figure 2).

**Figure 2:**
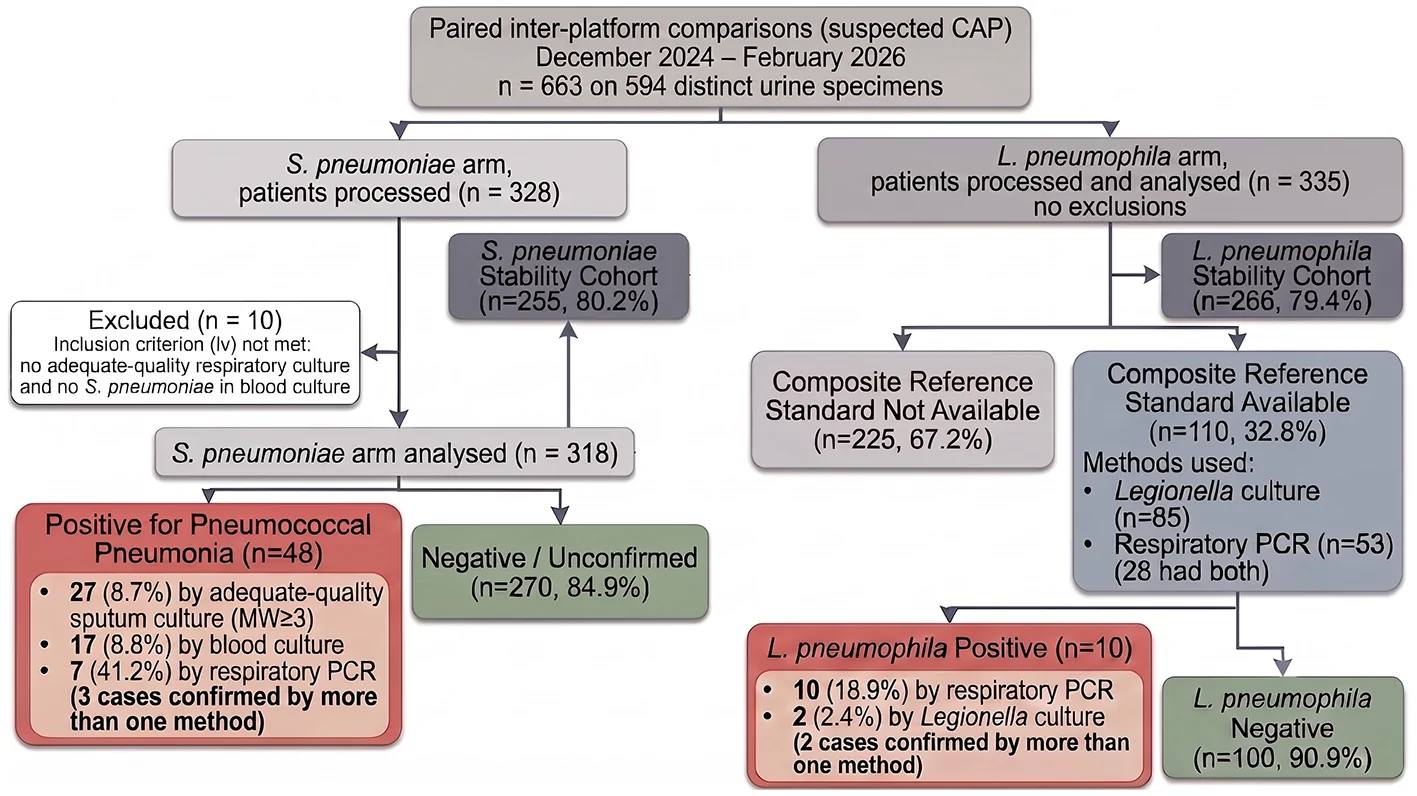
Patient inclusion and cohort stratification. A total of 663 paired inter-platform comparisons were performed on 594 distinct urine specimens from adult patients with suspected community-acquired pneumonia. Of these, 328 initial patients belonged to the *S. pneumoniae* arm. Ten patients were excluded because they failed to meet the specific reference standard inclusion criteria. The *L. pneumophila* arm accounted for 335 patients (no exclusions were recorded for this arm). Each arm was analyzed as a Total cohort and as a Stability cohort (specimens processed within 60 days of freezing). Within the reference-positive boxes, percentages are calculated among the patients in whom each reference test was performed, as in Table 1, and not as a proportion of the reference-positive group.

In the *S. pneumoniae* arm, the median storage interval prior to LIAISON^®^ testing was 30 days (IQR, 19–49; range, 4–407). Notably, 19.8% of these specimens were processed after more than 60 days and were therefore not included in the Stability cohort (*n* = 255). Furthermore, 12.6% of the specimens were processed after more than 180 days. In the *L. pneumophila* arm, the median interval was 31 days (IQR, 19–56; range, 1–376). The corresponding proportions for delayed processing were 20.6% (more than 60 days) and 8.4% (more than 180 days). The full interval distribution for both arms is presented in Supplementary Table S1.

In the *S. pneumoniae* arm, 48 of the 318 patients (15.1%) were confirmed positive for pneumococcal pneumonia according to the predefined composite reference standard. Specifically, 27 were diagnosed by adequate-quality sputum culture (Murray-Washington grade ≥3), 17 by blood culture, and 7 by respiratory PCR, with 3 cases testing positive by more than one method. A single reference method was performed in 130 patients (40.9%). Two methods were performed in 173 patients (54.4%). All three reference methods were performed in 15 patients (4.7%). Among the 270 reference-negative patients, these corresponding figures were 114 (42.2%), 148 (54.8%), and 8 (3.0%). The observed prevalence was lower than the 19% assumed a priori (Section 2.6). Nevertheless, the achieved precision for sensitivity (95% CI half-width ±12–13%) matched the pre-specified target.

In the *L. pneumophila* arm, *Legionella* culture and respiratory PCR were performed on 85 (25.4%) and 53 (15.8%) patients, respectively. Specifically, 57 patients underwent culture only. Another 25 patients underwent PCR testing only. Both diagnostic methods were performed concurrently on 28 patients. The composite reference standard (culture or PCR) was available for 32.8% of the patients (110/335), of which 9.1% (10/110) were positive. All 10 were positive by respiratory PCR and 2 of them were also positive by culture. The microbiological workup performed as part of routine clinical practice is summarized in Table 1.

**Table 1:** Specimen storage and microbiological workup, by study arm.

Variable	*L. pneumophila* (*n* = 335)	*S. pneumoniae* (*n* = 318)
Storage time before LIAISON^®^ testing, days, median (IQR)	31.0 (19.0–56.0)	30.0 (19.0–49.0)
Stability cohort ( ≤ 60 days), *n* (%)	266 (79.4%)	255 (80.2%)
Adequate-quality respiratory culture performed, *n* (%)	–	311 (97.8%)
Negative	–	206 (66.2%)^*^
*S. pneumoniae* isolated	–	27 (8.7%)^*^
Other microorganism isolated	–	78 (25.1%)^*^
Blood culture performed, *n* (%)	–	193 (60.7%)
Negative	–	171 (88.6%)^*^
*S. pneumoniae* isolated	–	17 (8.8%)^*^
Other microorganism isolated	–	5 (2.6%)^*^
*Legionella* culture performed, *n* (%)	85 (25.4%)	–
Negative	83 (97.6%)^*^	–
Positive	2 (2.4%)^*^	–
Respiratory PCR performed, *n* (%)	53 (15.8%)	17 (5.3%)
Negative	43 (81.1%)^*^	4 (23.5%)^*^
Positive	10 (18.9%)^*^	7 (41.2%)^*^
Other microorganism detected	–	6 (35.3%)
Composite reference standard available, *n* (%)	110 (32.8%)	318 (100.0%)
Negative	100 (90.9%)^*^	270 (84.9%)^*^
Positive	10 (9.1%)^*^	48 (15.1%)^*^

IQR, interquartile range. ^*^Percentage calculated among those in whom the test was performed.

### 3.2 Streptococcus pneumoniae

#### 3.2.1 Inter-platform agreement: total cohort

In the primary analysis, the positivity rate in the total cohort was 32.7% (95% CI, 27.8–38.0) for the Sofia^®^ *S. pneumoniae* FIA and 19.8% (95% CI, 15.8–24.5) for the LIAISON^®^ *S. pneumoniae* Ag assay. This difference was statistically significant (*p* < 0.001). The OPA was 85.2%, with a PPA and NPA (using the Sofia^®^ FIA as the comparator) of 57.7% and 98.6%, respectively. Cohen's kappa coefficient was 0.63. Forty-seven of 318 patients (14.8%) had discordant results: 44 were Sofia+/LIAISON– and 3 were Sofia–/LIAISON+. Among the 44 Sofia+/LIAISON– cases, 8 met the composite reference standard for pneumococcal pneumonia (6 by adequate-quality sputum culture, 1 by blood culture, and 1 by respiratory PCR). One of the 3 Sofia–/LIAISON+ discordant cases met the composite reference standard, by blood culture (Table 2).

**Table 2:** Inter-platform agreement between the LIAISON^®^ *S. pneumoniae* Ag assay and the Sofia^®^ *S. pneumoniae* FIA in the primary analysis, by cohort.

Parameter	Sofia^®^ FIA		
	Positive	Negative	Total
Total cohort (*n* = 318)			
LIAISON^®^ positive	60	3	**63**
LIAISON^®^ negative	44	211	**255**
Total	**104**	**214**	**318**
Discordant, *n* (%)	47 (14.8)		
OPA, % (95% CI)	85.2 (80.9–88.7)		
PPA, % (95% CI)	57.7 (48.1–66.7)		
NPA, % (95% CI)	98.6 (96.0–99.5)		
Cohen's kappa (95% CI)	0.63 (0.53–0.72)		
Positivity rate difference, pp	12.9		
Discordant pairs in the direction of the difference, *n*/*n* (%; 95% CI); *p*	44/47 (93.6; 82.5–98.7); *p* < 0.001		
Stability cohort (*n* = 255)			
LIAISON^®^ positive	54	2	**56**
LIAISON^®^ negative	34	165	**199**
Total	**88**	**167**	**255**
Discordant, *n* (%)	**36 (14.1)**		
OPA, % (95% CI)	85.9 (81.1–89.6)		
PPA, % (95% CI)	61.4 (50.9–70.9)		
NPA, % (95% CI)	98.8 (95.7–99.7)		
Cohen's kappa (95% CI)	0.66 (0.55–0.76)		
Positivity rate difference, pp	12.5		
Discordant pairs in the direction of the difference, *n*/*n* (%; 95% CI); *p*	34/36 (94.4; 81.3–99.3); *p* < 0.001		

CI, confidence interval; NPA, negative percent agreement; OPA, overall percent agreement; PPA, positive percent agreement. Bold type indicates cohort subheadings, row labels and marginal totals; it does not denote statistical significance.

After discrepancy resolution (secondary analysis), the positivity rate in the Total cohort was 28.9% (95% CI, 24.2–34.1) for the Sofia^®^ *S. pneumoniae* FIA and 19.8% (95% CI, 15.8–24.5) for the LIAISON^®^ *S. pneumoniae* Ag assay (*p* < 0.001). The OPA was 90.3%, with a PPA and NPA (using the Sofia^®^ FIA as the comparator) of 67.4% and 99.6%, respectively. Cohen's *kappa* coefficient was 0.74 (Table 3).

**Table 3:** Inter-platform agreement between the LIAISON^®^ *S. pneumoniae* Ag assay and the Sofia^®^ *S. pneumoniae* FIA after discrepancy resolution (secondary analysis), by cohort.

Parameter	Sofia^^®^^ FIA		
	Positive	Negative	Total
Total cohort (*n* = 318)			
LIAISON^®^ positive	62	1	**63**
LIAISON^®^ negative	30	225	**255**
Total	**92**	**226**	**318**
Discordant, *n* (%)	31 (9.7)		
OPA, % (95% CI)	90.3 (86.5–93.0)		
PPA, % (95% CI)	67.4 (57.3–76.1)		
NPA, % (95% CI)	99.6 (97.5–99.9)		
Cohen's kappa (95% CI)	0.74 (0.65–0.83)		
Positivity rate difference, pp	9.1		
Discordant pairs in the direction of the difference, *n*/*n* (%; 95% CI); *p*	30/31 (96.8; 83.3–99.9); *p* < 0.001		
Stability cohort (*n* = 255)			
LIAISON^®^ positive	55	1	**56**
LIAISON^®^ negative	24	175	**199**
Total	**79**	**176**	**255**
Discordant, *n* (%)	25 (9.8)		
OPA, % (95% CI)	90.2 (85.9–93.3)		
PPA, % (95% CI)	69.6 (58.8–78.7)		
NPA, % (95% CI)	99.4 (96.9–99.9)		
Cohen's kappa (95% CI)	0.75 (0.66–0.84)		
Positivity rate difference, pp	9.0		
Discordant pairs in the direction of the difference, *n*/*n* (%; 95% CI); *p*	24/25 (96.0; 79.6–99.9); *p* < 0.001		

CI, confidence interval; NPA, negative percent agreement; OPA, overall percent agreement; PPA, positive percent agreement. Bold type indicates cohort subheadings, row labels and marginal totals; it does not denote statistical significance.

Overall, 31 of 318 patients (9.7%) had discordant results: 30 were Sofia+/LIAISON– and 1 was Sofia–/LIAISON+. Among the 30 Sofia+/LIAISON– cases, 5 met the composite reference standard for pneumococcal pneumonia (3 by adequate-quality sputum culture, 1 by blood culture, and 1 by respiratory PCR). The single Sofia–/LIAISON+ discordant case did not meet the composite reference standard due to a lack of microbiological confirmation for pneumococcal pneumonia (Table 3).

#### 3.2.2 Sensitivity and specificity against the composite reference standard: total cohort

In the primary analysis, the Sofia^®^ FIA showed a sensitivity of 77.1%, a specificity of 75.2%, a PPV of 35.6% and an NPV of 94.9%. The LIAISON^®^ assay showed a sensitivity of 62.5%, a specificity of 87.8%, a PPV of 47.6% and an NPV of 92.9%. Positive and negative likelihood ratios were 3.11 and 0.30 for the Sofia^®^ FIA, and 5.11 and 0.43 for the LIAISON^®^ assay (Table 4).

**Table 4:** Diagnostic performance of the Sofia^®^ FIA and the LIAISON^®^ Ag assay for *S. pneumoniae* against the composite reference standard in the primary analysis, by cohort.

Parameter	Sofia^®^ FIA	LIAISON^®^ Ag	Difference, pp	Discordant pairs in the direction of the difference, *n*/*n* (%; 95% CI); *p*
Total cohort (*n* = 318)				
2 × 2 counts against the composite reference standard				
Assay-positive/reference-positive (*n* = 48)	37	30	–	–
Assay-negative/reference-positive	11	18	–	–
Assay-positive/reference-negative (*n* = 270)	67	33	–	–
Assay-negative/reference-negative	203	237	–	–
Accuracy estimates				
Sensitivity, % (95% CI)	77.1 (63.5–86.7)	62.5 (48.4–74.8)	14.6	8/9 (88.9; 51.8–99.7); *p* = 0.039
Specificity, % (95% CI)	75.2 (69.7–80.0)	87.8 (83.3–91.2)	12.6	36/38 (94.7; 82.3–99.4); *p* < 0.001
PPV, % (95% CI)	35.6 (27.0–45.1)	47.6 (35.8–59.7)	–	–
NPV, % (95% CI)	94.9 (91.0–97.1)	92.9 (89.1–95.5)	–	–
LR+ (95% CI)	3.11 (2.40–4.02)	5.11 (3.47–7.53)	–	–
30.6-7.4,-1.8498ptLR– (95% CI)	0.30 (0.18–0.51)	0.43 (0.30–0.62)	–	–
Stability cohort (*n* = 255)				
2 × 2 counts against the composite reference standard				
Assay-positive/reference-positive (*n* = 38)	33	27	–	–
Assay-negative/reference-positive	5	11	–	–
Assay-positive/reference-negative (*n* = 217)	55	29	–	–
Assay-negative/reference-negative	162	188	–	–
Accuracy estimates				
Sensitivity, % (95% CI)	86.8 (72.7–94.2)	71.1 (55.2–83.0)	15.8	6/6 (100; 54.1–100); *p* = 0.031
Specificity, % (95% CI)	74.7 (68.5–80.0)	86.6 (81.5–90.5)	12.0	28/30 (93.3; 77.9–99.2); *p* < 0.001
PPV, % (95% CI)	37.5 (28.1–47.9)	48.2 (35.7–61.0)	–	–
NPV, % (95% CI)	97.0 (93.2–98.7)	94.5 (90.4–96.9)	–	–
LR+ (95% CI)	3.43 (2.64–4.44)	5.32 (3.58–7.89)	–	–
LR– (95% CI)	0.18 (0.08–0.40)	0.33 (0.20–0.55)	–	–

CI, confidence interval; FIA, fluorescence immunoassay; LR+, positive likelihood ratio; LR–, negative likelihood ratio; NPV, negative predictive value; PPV, positive predictive value.

The paired sensitivity difference was 14.6 percentage points in favor of the Sofia^®^ FIA (8 of 9 discordant pairs; 88.9%, exact 95% CI 51.8–99.7; *p* = 0.039), and the paired specificity difference was 12.6 percentage points in favor of the LIAISON^®^ assay (36 of 38 discordant pairs; 94.7%, exact 95% CI 82.3–99.4; *p* < 0.001) (Table 4).

After discrepancy resolution (secondary analysis), the Sofia^®^ FIA showed a sensitivity of 72.9%, a specificity of 78.9%, a PPV of 38.0% and an NPV of 94.2%. Positive and negative likelihood ratios were 3.45 and 0.34. The LIAISON^®^ estimates are identical to those of the pre-resolution analysis, because the LIAISON^®^ assay was never repeated in this arm (Table 5).

**Table 5:** Diagnostic performance of the Sofia^®^ FIA and LIAISON^®^ Ag assays for *S. pneumoniae* against the composite reference standard after discrepancy resolution (secondary analysis), by cohort.

Parameter	Sofia^®^ FIA	LIAISON^®^ Ag	Difference, pp	Discordant pairs in the direction of the difference, *n*/*n* (%; 95% CI); *p*
Total cohort (*n* = 318)				
2 × 2 counts against the composite reference standard				
Assay-positive/reference-positive (*n* = 48)	35	30	–	–
Assay-negative/reference-positive	13	18	–	–
Assay-positive/reference-negative (*n* = 270)	57	33	–	–
Assay-negative/reference-negative	213	237	–	–
Accuracy estimates				
Sensitivity, % (95% CI)	72.9 (59.0–83.4)	62.5 (48.4–74.8)	10.4	5/5 (100; 47.8–100); *p* = 0.063
Specificity, % (95% CI)	78.9 (73.6–83.3)	87.8 (83.3–91.2)	8.9	25/26 (96.2; 80.4–99.9); *p* < 0.001
PPV, % (95% CI)	38.0 (28.8–48.3)	47.6 (35.8–59.7)	–	–
NPV, % (95% CI)	94.2 (90.4–96.6)	92.9 (89.1–95.5)	–	–
LR+ (95% CI)	3.45 (2.59–4.61)	5.11 (3.47–7.53)	–	–
30.6-7.4,-1.8498ptLR– (95% CI)	0.34 (0.21–0.55)	0.43 (0.30–0.62)	–	–
Stability cohort (*n* = 255)				
2 × 2 counts against the composite reference standard				
Assay-positive/reference-positive (*n* = 38)	30	27	–	–
Assay-negative/reference-positive	8	11	–	–
Assay-positive/reference-negative (*n* = 217)	49	29	–	–
Assay-negative/reference-negative	168	188	–	–
Accuracy estimates				
Sensitivity, % (95% CI)	78.9 (63.7–88.9)	71.1 (55.2–83.0)	7.9	3/3 (100; 29.2–100); *p* = 0.250
Specificity, % (95% CI)	77.4 (71.4–82.5)	86.6 (81.5–90.5)	9.2	21/22 (95.5; 77.2–99.9); *p* < 0.001
PPV, % (95% CI)	38.0 (28.1–49.0)	48.2 (35.7–61.0)	–	–
NPV, % (95% CI)	95.5 (91.3–97.7)	94.5 (90.4–96.9)	–	–
LR+ (95% CI)	3.50 (2.60–4.70)	5.32 (3.58–7.89)	–	–
LR– (95% CI)	0.27 (0.15–0.51)	0.33 (0.20–0.55)	–	–

CI, confidence interval; FIA, fluorescence immunoassay; LR+, positive likelihood ratio; LR–, negative likelihood ratio; NPV, negative predictive value; PPV, positive predictive value.

The paired sensitivity difference was 10.4 percentage points in favor of the Sofia^®^ FIA and was no longer statistically significant (*p* = 0.063), whereas the apparent specificity of the LIAISON^®^ assay exceeded that of the Sofia^®^ FIA by 8.9 percentage points (*p* < 0.001) (Table 5).

A descriptive breakdown of cases stratified by reference method is provided in Supplementary Table S2. The Sofia^®^ FIA detected an equal or higher number of confirmed cases across each individual reference standard compared to the LIAISON^®^ assay.

Seven patients were classified as reference-positive by respiratory PCR alone. None was positive by the LIAISON^®^ assay and one was positive by the Sofia^®^ FIA, in both analyses. In these seven patients the LIAISON^®^ index ranged from 0.23 to 0.44, against a positivity cut-off of 1.00, with a median of 0.27 (IQR, 0.25–0.41). The corresponding median was 2.72 (IQR, 0.73–16.60) in the 41 culture-confirmed patients and 0.37 (IQR, 0.30–0.51) in the 270 reference-negative patients.

#### 3.2.3 Inter-platform agreement: stability cohort

In the primary analysis, the positivity rate in the stability cohort was 34.5% (95% CI, 28.9–40.5) for the Sofia^®^ *S. pneumoniae* FIA and 22.0% (95% CI, 17.3–27.4) for the LIAISON^®^ *S. pneumoniae* Ag assay (*p* < 0.001). The OPA was 85.9%, with a PPA of 61.4% and an NPA of 98.8%. Cohen's kappa coefficient was 0.66. Thirty-six of 255 patients (14.1%) had discordant results: 34 were Sofia+/LIAISON– and 2 were Sofia–/LIAISON+. Of the 34 Sofia+/LIAISON– discordant cases, 6 met the composite reference standard for pneumococcal pneumonia (confirmed by adequate-quality sputum culture in 5 cases and blood culture in 1). Neither of the 2 Sofia–/LIAISON+ discordant cases met the composite reference standard (Table 2).

After discrepancy resolution (secondary analysis), within the Stability cohort ( ≤ 60 days), the Sofia^®^ *S. pneumoniae* FIA showed a significantly higher positivity rate than the LIAISON^®^ *S. pneumoniae* Ag assay (31.0% (95% CI: 25.6–36.9) vs. 22.0% (95% CI: 17.3–27.4); *p* < 0.001). The OPA was 90.2%, reflecting a PPA of 69.6% and an NPA of 99.4%. Cohen's kappa coefficient was 0.75 (Table 3).

In this cohort, 25 of 255 patients (9.8%) had discordant results: 24 were Sofia+/LIAISON– and 1 was Sofia–/LIAISON+. Of the 24 Sofia+/LIAISON– discordant cases, 3 met the composite reference standard for pneumococcal pneumonia (confirmed by adequate-quality sputum culture in 2 cases and blood culture in 1). The single Sofia–/LIAISON+ discordant case did not meet the composite reference standard (Table 3).

#### 3.2.4 Sensitivity and specificity against the composite reference standard: stability cohort

In the primary analysis, the Sofia^®^ FIA showed a sensitivity of 86.8%, a specificity of 74.7%, a PPV of 37.5% and an NPV of 97.0%. The LIAISON^®^ assay showed a sensitivity of 71.1%, a specificity of 86.6%, a PPV of 48.2% and an NPV of 94.5%. Positive and negative likelihood ratios were 3.43 and 0.18 for the Sofia^®^ FIA, and 5.32 and 0.33 for the LIAISON^®^ assay.

The paired sensitivity difference was 15.8 percentage points in favor of the Sofia^®^ FIA (*p* = 0.031), and the paired specificity difference was 12.0 percentage points in favor of the LIAISON^®^ assay (*p* < 0.001) (Table 4).

After discrepancy resolution (secondary analysis), the Sofia^®^ FIA showed a sensitivity of 78.9%, a specificity of 77.4%, a PPV of 38.0% and an NPV of 95.5%. Positive and negative likelihood ratios were 3.50 and 0.27.

The paired sensitivity difference was 7.9 percentage points in favor of the Sofia^®^ FIA and was not statistically significant (*p* = 0.250), whereas the apparent specificity of the LIAISON^®^ assay exceeded that of the Sofia^®^ FIA by 9.2 percentage points (*p* < 0.001) and its positivity rate was 9.0 percentage points lower (*p* < 0.001) (Table 5).

#### 3.2.5 Restricted analysis of the composite reference standard

A patient was reference-positive only if *S. pneumoniae* was isolated from blood culture or from an adequate-quality respiratory culture, irrespective of which other reference tests were performed. A patient was reference-negative only if both a blood culture and an adequate-quality respiratory culture had been performed and neither yielded *S. pneumoniae*. This restriction excluded seven patients classified as positive by respiratory PCR alone and 114 reference-negative patients with an incomplete culture work-up. It left 197 patients in the Total cohort, comprising 41 reference-positive and 156 reference-negative patients, and 161 patients in the Stability cohort, comprising 37 reference-positive and 124 reference-negative patients.

In the Total cohort, sensitivity was 87.8% for the Sofia^®^ FIA and 73.2% for the LIAISON^®^ assay. The paired difference of 14.6 percentage points was not statistically significant (7 of 8 discordant pairs, 87.5%, *p* = 0.070). After resolution (secondary analysis), sensitivity was 82.9% for the Sofia^®^ FIA, and the paired difference of 9.8 percentage points was not statistically significant (4 of 4 discordant pairs, *p* = 0.125).

Apparent specificity was 74.4% for the Sofia^®^ FIA and 87.8% for the LIAISON^®^ assay in the primary analysis, a difference of 13.5 percentage points (22 of 23 discordant pairs, *p* < 0.001). After resolution the corresponding values were 76.9% and 87.8%, a difference of 10.9 percentage points (17 of 17 discordant pairs, *p* < 0.001).

In the Stability cohort, sensitivity was 89.2% for the Sofia^®^ FIA and 73.0% for the LIAISON^®^ assay, and the paired difference of 16.2 percentage points remained statistically significant (6 of 6 discordant pairs, *p* = 0.031). After discrepancy resolution (secondary analysis), the difference of 8.1 percentage points was not statistically significant (*p* = 0.250). The specificity difference favored the LIAISON^®^ assay in both analyses (12.1 and 10.5 percentage points, *p* < 0.001). The complete estimates are presented in Supplementary Table S4.

### 3.3 Legionella pneumophila

#### 3.3.1 Inter-platform agreement: total cohort

In the primary analysis, the positivity rate in the total cohort was 15.2% (95% CI, 11.8–19.5) for the Sofia^®^ *Legionella* FIA and 11.6% (95% CI, 8.6–15.5) for the LIAISON^®^ *Legionella* Urinary Ag assay (*p* = 0.017). Using the Sofia^®^ FIA as the comparator, the OPA was 93.4%, with a PPA of 66.7% and an NPA of 98.2%. Cohen's kappa coefficient was 0.72. Among the 10 cases confirmed by the composite reference standard, the Sofia^®^ FIA was positive in 7 and the LIAISON^®^ assay in 6.

Twenty-two of 335 patients (6.6%) had discordant results: 17 were Sofia+/LIAISON– and 5 were Sofia–/LIAISON+ (Table 6). Among the 17 Sofia+/LIAISON– samples, a composite reference standard was available for 10, two were positive (both confirmed by respiratory PCR) and 8 were negative. The remaining 7 samples lacked culture or PCR data. Among the 5 Sofia–/LIAISON+ samples, a composite reference standard was available for 1, which was positive (confirmed by respiratory PCR). The remaining 4 samples lacked reference standard results (Table 6).

**Table 6:** Inter-platform agreement between the LIAISON^®^ *Legionella* Urinary Ag assay and the Sofia^®^ *Legionella* FIA in the primary analysis, by cohort.

Parameter	Sofia^®^ FIA		
	Positive	Negative	Total
Total cohort (*n* = 335)			
LIAISON^®^ positive	34	5	**39**
LIAISON^®^ negative	17	279	**296**
Total	**51**	**284**	**335**
Discordant, *n* (%)	22 (6.6)		
OPA, % (95% CI)	93.4 (90.3–95.6)		
PPA, % (95% CI)	66.7 (53.0–78.0)		
NPA, % (95% CI)	98.2 (95.9–99.2)		
Cohen's kappa (95% CI)	0.72 (0.60–0.83)		
Positivity rate difference, pp	3.6		
Discordant pairs in the direction of the difference, *n*/*n* (%; 95% CI); *p*	17/22 (77.3; 54.6–92.2); *p* = 0.017		
Stability cohort (*n* = 266)			
LIAISON^®^ positive	17	2	**19**
LIAISON^®^ negative	11	236	**247**
Total	**28**	**238**	**266**
Discordant, *n* (%)	13 (4.9)		
OPA, % (95% CI)	95.1 (91.8–97.1)		
PPA, % (95% CI)	60.7 (42.4–76.4)		
NPA, % (95% CI)	99.2 (97.0–99.8)		
Cohen's kappa (95% CI)	0.70 (0.54–0.86)		
Positivity rate difference, pp	3.4		
Discordant pairs in the direction of the difference, *n*/*n* (%; 95% CI); *p*	11/13 (84.6; 54.6–98.1); *p* = 0.023		

CI, confidence interval; NPA, negative percent agreement; OPA, overall percent agreement; PPA, positive percent agreement. Bold type indicates cohort subheadings, row labels and marginal totals; it does not denote statistical significance.

After heat treatment and re-analysis of the supernatant on both platforms (secondary analysis), the positivity rate for the Sofia^®^ *Legionella* FIA remained higher than that of the LIAISON^®^ *Legionella* Urinary Ag assay (14.6% (95% CI: 11.2–18.8) vs. 11.3% (95% CI: 8.4–15.2); *p* = 0.007). The OPA was 95.5%, reflecting a PPA of 73.5% and an NPA of 99.3%. Cohen's kappa coefficient was 0.80 (Table 7).

**Table 7:** Inter-platform agreement between the LIAISON^®^ *Legionella* Urinary Ag assay and the Sofia^®^ *Legionella* FIA after heat treatment and re-analysis of the supernatant (secondary analysis), by cohort.

Parameter	Sofia^®^ FIA		
	**Positive**	**Negative**	**Total**
Total cohort (*n* = 335)			
LIAISON^®^ positive	36	2	**38**
LIAISON^®^ negative	13	284	**297**
Total	**49**	**286**	**335**
Discordant, *n* (%)	15 (4.5)		
OPA, % (95% CI)	95.5 (92.7–97.3)		
PPA, % (95% CI)	73.5 (59.7–83.8)		
NPA, % (95% CI)	99.3 (97.5–99.8)		
Cohen's kappa (95% CI)	0.80 (0.70–0.90)		
Positivity rate difference, pp	3.3		
Discordant pairs in the direction of the difference, *n*/*n* (%; 95% CI); *p*	13/15 (86.7; 59.5–98.3); *p* = 0.007		
Stability cohort (*n* = 266)			
LIAISON^®^ positive	18	1	**19**
LIAISON^®^ negative	7	240	**247**
Total	**25**	**241**	**266**
Discordant, *n* (%)	8 (3.0)		
OPA, % (95% CI)	97.0 (94.2–98.5)		
PPA, % (95% CI)	72.0 (52.4–85.7)		
NPA, % (95% CI)	99.6 (97.7–99.9)		
Cohen's kappa (95% CI)	0.80 (0.67–0.94)		
Positivity rate difference, pp	2.3		
Discordant pairs in the direction of the difference, *n*/*n* (%; 95% CI); *p*	7/8 (87.5; 47.3–99.7); *p* = 0.070		

CI, confidence interval; NPA, negative percent agreement; OPA, overall percent agreement; PPA, positive percent agreement. Bold type indicates cohort subheadings, row labels and marginal totals; it does not denote statistical significance.

Among the 10 cases confirmed by the composite reference standard, the Sofia^®^ FIA was positive in 8 and the LIAISON^®^ assay in 6. In this study arm, 15 of 335 patients (4.5%) had discordant results: 13 were Sofia+/LIAISON– and 2 were Sofia–/LIAISON+. Among the 13 Sofia+/LIAISON– cases, a composite reference standard was available for 7, two were positive (both confirmed by respiratory PCR) and 5 were negative. The remaining six cases lacked culture or PCR data. Neither of the 2 Sofia–/LIAISON+ cases had reference standard results available (Table 7).

#### 3.3.2 Inter-platform agreement: stability cohort

In the main analysis, the positivity rate in the stability cohort was 10.5% (95% CI, 7.4–14.8) for the Sofia^®^ *Legionella* FIA and 7.1% (95% CI, 4.6–10.9) for the LIAISON^®^ *Legionella* Urinary Ag assay (*p* = 0.023) in the Stability cohort. The OPA was 95.1%, with a PPA of 60.7% and an NPA of 99.2%. Cohen's kappa coefficient was 0.70. Thirteen of 266 patients (4.9%) had discordant results: 11 were Sofia+/LIAISON– and 2 were Sofia–/LIAISON+ (Table 6).

Among the 11 Sofia+/LIAISON– cases, a composite reference standard was available for 7 (all negative); the remaining 4 lacked reference data. Neither of the 2 Sofia–/LIAISON+ cases had reference standard results available (Table 6).

After heat treatment and re-analysis (secondary analysis), the positivity rate was 9.4% (95% CI, 6.4–13.5) for the Sofia^®^ *Legionella* FIA and 7.1% (95% CI, 4.6–10.9) for the LIAISON^®^ *Legionella* Urinary Ag assay. No statistically significant difference was observed between the two assays (*p* = 0.070). The OPA was 97.0%, with a PPA of 72.0% and an NPA of 99.6%. Cohen's kappa coefficient was 0.80 (Table 7).

In this cohort, 8 of 266 patients (3.0%) had discordant results: 7 were Sofia+/LIAISON– and 1 was Sofia–/LIAISON+. Among the 7 Sofia+/LIAISON– cases, a composite reference standard was available for 4 (all negative); the remaining 3 lacked reference data. The single Sofia–/LIAISON+ case also lacked reference standard results (Table 7).

A descriptive breakdown of the reference-confirmed cases by reference method is provided in Supplementary Table S3. Consistent with the rationale stated in Section 2.6, these are descriptive counts and no accuracy estimates were derived for this arm.

### 3.4 Discrepancy resolution

In the *S. pneumoniae* arm, 47 of 318 patients (14.8%) had discordant results between platforms on first testing: 44 were Sofia-positive/LIAISON-negative and 3 were Sofia-negative/LIAISON-positive. All entered the discrepancy-resolution protocol, in which the Sofia^®^ FIA was repeated on the same thawed aliquot used for LIAISON^®^ testing. Sixteen discordances (34.0%) were resolved, 14 to a concordant-negative and 2 to a concordant-positive result, whereas 31 (66.0%) persisted and are those reported in Section 3.2.1.

In the *L. pneumophila* arm, 22 of 335 patients (6.6%) had initially discordant results: 17 Sofia-positive/LIAISON-negative and 5 Sofia-negative/LIAISON-positive. After heat treatment and re-analysis of the supernatant on both platforms, 7 discordances (31.8%) were resolved, 5 to a concordant-negative and 2 to a concordant-positive result, and 15 (68.2%) persisted. The effect of the discrepancy-resolution protocol on each agreement measure is quantified in Supplementary Table S5.

## 4 Discussion

To the best of our knowledge, no previous study has provided a head-to-head comparison between the fully automated LIAISON^®^ CLIA and the Sofia^®^ FIA for *S. pneumoniae* urinary antigen detection. Furthermore, diagnostic accuracy in this arm was evaluated against a composite microbiological reference standard. Crucially, this reference standard was entirely independent of any urinary antigen testing. In the primary analysis, the two assays showed substantial concordance. Cohen's kappa was 0.63-0.66 for *S. pneumoniae* and 0.70–0.72 for *L. pneumophila*. The negative percent agreement (NPA) was consistently high (≥98% across all cohorts), whereas the positive percent agreement (PPA) was substantially lower (< 74%) in both arms.

Regarding secondary endpoints, apparent sensitivity favored the Sofia^®^ FIA in every comparison, significantly so only before resolution (Total 77.1% *vs*. 62.5%, *p* = 0.039; Stability 86.8% vs. 71.1%, *p* = 0.031), and not after it (*p* ≥ 0.063) or in the *post-hoc* culture-restricted Total cohort (*p* = 0.070). Because the primary comparison places the Sofia^®^ FIA on fresh urine and the LIAISON^®^ assay on a frozen–thawed aliquot, this difference cannot be separated from the difference in specimen handling. In both analyses the LIAISON^®^ assay showed higher apparent specificity for *S. pneumoniae* and consistently returned lower positivity rates for both pathogens. The pneumococcal sensitivities observed in this study fall within ranges previously reported for UATs. Meta-analyses report a pooled sensitivity of approximately 66% (Yasuo et al., 2022) and 68.5% against culture (Sinclair et al., 2013). Similarly, a review of several post-2013 studies places real-world sensitivity between 60% and 65% (Hyams et al., 2020).

One key pre-analytical factor likely drives the lower sensitivity observed within our total cohort relative to the Stability cohort. Specifically, prolonged frozen storage may progressively degrade the target antigen. This degradation hypothesis fully aligns with the higher diagnostic sensitivity observed in the Stability cohort. However, because this cohort represents a nested subgroup, these data alone cannot definitively establish a causal storage effect.

The most common pattern of discordance was a Sofia-positive/LIAISON-negative result, accounting for 44 of 47 discordant cases in the Total cohort and 34 of 36 in the Stability cohort. This discrepancy explains both the higher positivity rate of the Sofia^®^ FIA and its lower apparent specificity (75.2% vs. 87.8% in the Total cohort; *p* < 0.001).

Expressed in a prevalence-independent format, this performance translates to a positive likelihood ratio of 5.11 for the LIAISON^®^ assay and 3.11 for the Sofia^®^ FIA within the Total cohort. The corresponding negative likelihood ratios for the two platforms were 0.43 and 0.30, respectively. Consequently, the difference between platforms is confined to the rule-in direction, directly mirroring the gap in specificity. Both assays remain similarly limited when a negative result is used to rule out disease.

Mechanistically, this difference cannot be attributed to different antigenic targets, as both assays detect the same analyte: the pneumococcal cell-wall C-polysaccharide (CWPS). This shared analyte is also present in other *Streptococcus mitis* group organisms (notably *S. mitis* and *S. oralis*), and cross-reactivity has been documented with the *S. anginosus* group (Hyams et al., 2020; Yu et al., 2022). Consequently, several factors may explain why a positive result does not reflect the true etiology of acute pneumonia. These include oropharyngeal colonization, non-pneumococcal streptococcal infections, recent vaccination, or recent pneumococcal disease, where antigenuria can persist for weeks (Hyams et al., 2020; Menéndez et al., 2020; Kim et al., 2022). This false-positive result, driven by cross-reactivity or antigen persistence, is particularly frequent in colonized patients with chronic obstructive pulmonary disease (Briones et al., 2006).

These diagnostic differences become critically apparent at the point of clinical decision-making. Within our cohort, the observed baseline prevalence was 15%. At this specific prevalence, a positive result raises the post-test probability to approximately 35% with the Sofia^®^ FIA and 47% with the LIAISON^®^ assay. Conversely, a negative result only lowers this probability to approximately 5% and 7%, respectively. Consequently, at prevalences typical of unselected CAP, neither assay functions as a decisive stand-alone rule-in or rule-out test. Therefore, clinicians should follow clinical practice guidelines to maintain a high pre-test probability for UATs (Metlay et al., 2019; Kim et al., 2022).

However, these findings must be interpreted cautiously because the composite reference standard is inherently insensitive. Blood cultures are positive in only 15–30% of pneumococcal pneumonia cases, and sputum cultures yield positive results in less than 60% of cases (Burgos et al., 2018; Hyams et al., 2020). Consequently, some unconfirmed Sofia-positive/LIAISON-negative results may represent true infections missed by culture rather than false positives. If so, the seemingly higher specificity of the LIAISON^®^ assay could partly reflect lower sensitivity. This possibility is supported by literature. When latent-class modeling is used to account for imperfect reference standards (Butler et al., 2003), the estimated specificity of CWPS-based urinary tests rises from approximately 84% to 97%, and sensitivity increases from 68% to 74% (Sinclair et al., 2013). Therefore, the true specificity of both platforms is likely higher than our culture-based estimates suggest, and the actual difference between them may be narrower.

This aligns with a previous Spanish CAP cohort, where the Sofia^®^ FIA detected more positive samples than the BinaxNOW^®^ test among culture-negative patients (Burgos et al., 2018). That study attributed the finding to greater sensitivity, highlighting that extra positive results from a sensitive assay should not automatically be dismissed as false positives.

The composite reference standard is imperfect, and its molecular component cannot distinguish infection from upper airway colonization. To assess the impact of this limitation, we repeated the accuracy analysis with the reference-positive stratum restricted to culture-confirmed cases and the reference-negative stratum restricted to patients with both respiratory and blood negative cultures. Under these strict criteria, all specificity comparisons were fully preserved. However, the sensitivity difference reached statistical significance only within the Stability cohort. Ultimately, this methodological restriction mitigates, but cannot entirely eliminate, the biases inherent to an imperfect reference standard.

For *L. pneumophila*, the negative percent agreement was high in both cohorts (κ = 0.70–0.72; NPA > 98% in all cohorts), indicating that the LIAISON^®^ assay was negative in almost all Sofia-negative patients. This aligns with the excellent agreement reported between the Sofia^®^ FIA and other *Legionella* UATs (κ = 0.97 against an immunochromatographic comparator) (Congestrì et al., 2019), as well as the consistently high specificity (90–100%) typical of lateral-flow *Legionella* assays (Diederen and Peeters, 2006; Wong et al., 2021b).

The reference standard was available for only 110 of the 335 patients (32.8%). Among these, just 10 cases were reference-confirmed. Specifically, the Sofia^®^ FIA was positive in 7 of these confirmed cases. The LIAISON^®^ assay was positive in only 6. Three of the confirmed cases were discordant and all three were PCR-confirmed: two were Sofia-positive/LIAISON-negative and one was Sofia-negative/LIAISON-positive. The two Sofia-positive/LIAISON-negative specimens had been stored for 72 and 300 days, which placed them outside the Stability cohort.

In the *L. pneumophila* arm, the positivity rate differed significantly between platforms in the Total and the Stability cohort in the primary analysis, and also in the Total cohort after discrepancy resolution. However, this difference was not statistically significant in the Stability cohort in this sensitivity analyses. The Stability cohort is a nested subset of the Total cohort, comprising 266 of the 335 patients. Consequently, this shift in statistical significance is more consistent with reduced statistical power than with a true storage-related effect.

Furthermore, the CLSI EP12 target of at least 60 comparator-positive specimens was not reached in either analysis of this arm. Prior to heat treatment, there were only 51 such specimens in the Total cohort and 28 in the Stability cohort. Following heat treatment, these counts decreased to 49 in the Total cohort and 25 in the Stability cohort. The resulting imprecision is clearly reflected in the confidence intervals for positive percent agreement (PPA). Taken together, these observations indicate a lower detection rate by the LIAISON^®^ assay relative to the Sofia^®^ FIA. Notably, no accuracy estimates were computed for this specific arm. Therefore, these figures represent measures of agreement with a non-reference comparator rather than true clinical sensitivity. Ultimately, the available data are too imprecise to support a definitive conclusion.

The three published evaluations (Ferraiuolo et al., 2025; Cusato et al., 2026; Azzato et al., 2026) report higher figures than our observed positive percent agreement. In our study, this agreement was 66.7% in the primary analysis and 73.5% after heat treatment and re-analysis of discrepant specimens. Ferraiuolo et al. (2025) reported a positive percent agreement of 98.0% (50/51) against concordant Binax™ and RIDASCREEN^®^ results. Cusato *et al*. (2026) reported agreements of 94.7% and 96.1% against Binax™ and RIDASCREEN^®^, respectively. Azzato et al. (2026) reported a figure of 86.3% against a Binax™ enzyme immunoassay. This latter study explicitly designated the Binax™ assay as its reference standard and reported this value as clinical sensitivity. In every study, including our own, the comparator was inherently an antigen test rather than an independent reference standard. Consequently, all these figures represent agreement estimates conditional on the specific comparator used.

The published evaluations differ from our study regarding comparator-positive stratum assembly and specimen composition. Both methodological differences inherently drive agreement in the same direction. Specifically, Ferraiuolo et al. (2025) required concordant Binax™ and RIDASCREEN^®^ positivity using leftover and contrived specimens. Cusato et al. (2026) strictly excluded four specimens that remained unclassifiable after repeat testing. Furthermore, 149 of their 307 samples originated from healthy subjects or patients with urinary tract infections. These highly controlled designs are appropriate for characterizing analytical specificity. Nevertheless, they actively concentrate the positive stratum on unambiguously classified cases. In contrast, our cohort exclusively comprised adults with suspected CAP. Most of these patients remained microbiologically unexplained. This broader clinical spectrum inherently captures more determinations near the decision threshold of each assay. Therefore, a lower agreement estimate is expected in our series based on study design alone. Ultimately, our findings and the higher published values are complementary rather than contradictory.

Another constraint specific to legionellosis is the difficulty of obtaining high-quality lower respiratory specimens, combined with the low and variable yield of *Legionella* culture (Pierre et al., 2017). These challenges explain why so few cases met our reference standard, and they underscore why rapid, non-invasive urinary testing is clinically essential. However, it is important to note that antigen tests can return false-negative results in early or mild disease (Menéndez et al., 2020). This study has several limitations, though we took specific steps to mitigate them:

First, the design was retrospective and pre-analytically asymmetric. The Sofia^®^ result was generated on fresh urine in routine practice, whereas the LIAISON^®^ result was obtained later on a frozen-thawed aliquot of the same specimen, so platform and specimen handling differ simultaneously in the primary analysis. Every specimen underwent exactly one freeze-thaw cycle, storage times were recorded, and a Stability cohort ( ≤ 60 days) was analyzed alongside the Total cohort. Differences between these nested cohorts are consistent with a storage effect but do not prove one, and absolute sensitivity may be underestimated relative to routine practice.

Second, the composite reference standard is imperfect and was applied heterogeneously. Blood culture, adequate-quality sputum culture and respiratory PCR were combined to increase case capture, but verification was differential: among the 270 reference-negative patients, one investigation was performed in 114 (42.2%), two in 148 (54.8%) and three in 8 (3.0%). Furthermore, seven patients entered the reference-positive stratum on a qualitative molecular result alone, with no cycle threshold available to separate colonization from infection, and data on prior antibiotic therapy were unavailable. An antigen-positive result with a negative reference investigation may therefore reflect an infection missed by the reference standard rather than a false positive. Consequently, all estimates where reported as apparent accuracy conditional on this standard. The restricted analysis in Section 3.2.5 addresses both features and preserved every between-platform comparison, but it cannot correct an imperfect reference standard.

Third, the comparison is between qualitative classifications. Initial Sofia^®^ indices were retrievable for only 7 patients in the *S. pneumoniae* arm and 5 in the *L. pneumophila* arm, because the analyzer did not retain historical numeric records. We could therefore not determine whether discordant results lay close to the decision threshold of both assays.

Fourth, the overall number of reference-confirmed patients remained low, particularly within the *L. pneumophila* arm. Consequently, inter-platform agreement served as the primary endpoint and explicitly dictated the study's sample size. Formal accuracy comparisons were restricted exclusively to *S. pneumoniae*.

Fifth, the *L. pneumophila* cohort was enriched by design, and candidate confirmed cases were identified in part on the basis of a positive Sofia^®^ FIA routine urinary antigen test. This tends to favor the Sofia^®^ FIA in the head-to-head comparison and to lower the apparent positive percent agreement of the LIAISON^®^ assay, and it cannot be corrected analytically. Excluding the routine result from the reference standard prevents incorporation bias in the accuracy analysis, but it does not address this selection bias in the agreement analysis. Cohen's kappa and percent agreement in this arm are additionally affected by the selected spectrum and prevalence of positive cases and do not transfer to an unselected CAP population.

Finally, this was a single-center study. However, both assays were evaluated on the same real-world samples in a single, high-volume laboratory. Using a uniform pre-analytical and discrepancy-resolution protocol (including the boiling step) eliminated the inter-center and inter-operator variability that could otherwise confound a head-to-head comparison. Additionally, the specific serogroup of the confirmed *L. pneumophila* cases was not identified. The Sofia^®^ FIA targets *L. pneumophila* serogroup 1, whereas the LIAISON^®^ assay also targets the peptidoglycan-associated lipoprotein and is intended to detect additional serogroups and species (Ferraiuolo et al., 2025). Non-serogroup 1 infections would therefore be expected to be missed by the Sofia^®^ FIA rather than by the LIAISON^®^ assay, and cannot account for the direction of the discordances observed.

In conclusion, the fully automated LIAISON^®^ CLIA demonstrated substantial agreement with the Sofia^®^ FIA for both *S. pneumoniae* and *L. pneumophila* urinary antigens. Although negative percent agreement exceeded 98% across all analyses, overall positive percent agreement was substantially lower (< 74%). Regarding *S. pneumoniae*, the LIAISON^®^ assay yielded a consistently lower overall positivity rate, and this profile drove its higher apparent specificity against the composite reference standard. Furthermore, its apparent sensitivity was lower than that of the Sofia^®^ FIA in the *S. pneumoniae* arm. Following discrepancy resolution, the observed difference in sensitivity failed to reach statistical significance in both cohorts. Ultimately, a prospective evaluation featuring simultaneous testing of fresh specimens is strictly required before these platforms can be considered interchangeable.

## Data Availability

The complete anonymised datasets underlying all analyses are provided as Supplementary Material. [Data Sheet 1] contains the record-level dataset for each study arm (Data S1, *S. pneumoniae*; Data S2, *L. pneumophila*), each with a variable dictionary, the reagent lot, calibration identifier, analyser serial number and assay cut-off applying to each determination, and a specimen-flow sheet. [Table 1] and [Table 2] provide the corresponding source data tables. Any further enquiries can be directed to the corresponding author.

## Supplementary material

Data Sheet 1 Anonymised record-level datasets underlying all analyses: Data S1 (*S. pneumoniae* arm) and Data S2 (*L. pneumophila* arm). Each includes a variable dictionary, the full record-level dataset and a specimen-flow sheet.

Table S1 Source data table for the *L. pneumophila* arm, from which Data S2 was compiled.

Table S2 Source data table for the *S. pneumoniae* arm, from which Data S1 was compiled.
**Table S3**
Supplementary Tables S1 to S5, as cited in the article text.
