# Supplementary material for: Inter-platform agreement and diagnostic performance of the automated LIAISON® chemiluminescence immunoassay vs. the Sofia® fluorescence immunoassay for Streptococcus pneumoniae and Legionella pneumophila urinary antigen detection
Source: Front Microbiol. 2026 Sep 21;17:1926157. doi: 10.3389/fmicb.2026.1926157 (PMC13635099; doi:10.3389/fmicb.2026.1926157)
Supplement: Supplementary file 1 [file Data_Sheet_1.docx]

**Supplementary Table S1.** Distribution of storage time before LIAISON® testing

| **Parameter** | ***S. pneumoniae arm* (n = 318)** | ***L. pneumophila* arm (n = 335)** |
| --- | --- | --- |
| Median, days | 30 | 31 |
| Interquartile range, days | 19–49 | 19–56 |
| Range, days | 4–407 | 1–376 |
| Stored ≤60 days (Stability cohort), n (%) | 255 (80.2) | 266 (79.4) |
| Stored >60 days, n (%) | 63 (19.8) | 69 (20.6) |
| Stored >180 days, n (%) | 40 (12.6) | 28 (8.4) |

*Storage time is the interval between the routine Sofia® test, performed on fresh urine, and LIAISON® testing. Every specimen underwent exactly one freeze–thaw cycle.*

**Supplementary Table S2.** Detection of reference-confirmed pneumococcal cases, stratified by reference method

| **Reference method** | **n** | **Pre-resolution** | | **After discrepancy resolution** | |
| --- | --- | --- | --- | --- | --- |
|  |  | **Sofia® FIA positive, n (%; 95% CI)** | **LIAISON® CLIA positive, n (%; 95% CI)** | **Sofia® FIA positive, n (%; 95% CI)** | **LIAISON® CLIA positive, n (%; 95% CI)** |
| Blood culture | 17 | 15 (88.2; 65.7–96.7) | 15 (88.2; 65.7–96.7) | 16 (94.1; 73.0–99.0) | 15 (88.2; 65.7–96.7) |
| Adequate-quality sputum culture (Murray–Washington ≥3) | 27 | 24 (88.9; 71.9–96.1) | 18 (66.7; 47.8–81.4) | 21 (77.8; 59.2–89.4) | 18 (66.7; 47.8–81.4) |
| Respiratory PCR | 7 | 1 (14.3; 2.6–51.3) | 0 (0.0; 0.0–35.4) | 1 (14.3; 2.6–51.3) | 0 (0.0; 0.0–35.4) |
| Any method (composite reference standard) | 48 | 37 (77.1; 63.5–86.7) | 30 (62.5; 48.4–74.8) | 35 (72.9; 59.0–83.4) | 30 (62.5; 48.4–74.8) |

*Categories are not mutually exclusive: three cases were positive by more than one method. Percentages are calculated within each method-specific stratum; 95% confidence intervals are Wilson intervals. Stratified estimates are descriptive only and were not compared statistically, because the number of cases identified by each individual method is too small to support stable estimates.*

**Supplementary Table S3.** Detection of reference-confirmed *Legionella pneumophila* cases, stratified by reference method

| **Reference method** | **n** | **Before heat treatment** | | **After heat treatment and re-analysis** | |
| --- | --- | --- | --- | --- | --- |
|  |  | **Sofia® FIA positive, n (%; 95% CI)** | **LIAISON® CLIA positive, n (%; 95% CI)** | **Sofia® FIA positive, n (%; 95% CI)** | **LIAISON® CLIA positive, n (%; 95% CI)** |
| Respiratory culture | 2 | 1 (50.0; 9.5–90.5) | 1 (50.0; 9.5–90.5) | 1 (50.0; 9.5–90.5) | 1 (50.0; 9.5–90.5) |
| Respiratory PCR | 10 | 7 (70.0; 39.7–89.2) | 6 (60.0; 31.3–83.2) | 8 (80.0; 49.0–94.3) | 6 (60.0; 31.3–83.2) |
| Any method (composite reference standard) | 10 | 7 (70.0; 39.7–89.2) | 6 (60.0; 31.3–83.2) | 8 (80.0; 49.0–94.3) | 6 (60.0; 31.3–83.2) |

*Categories are not mutually exclusive: both culture-confirmed cases were also PCR-confirmed, so the composite reference standard comprises the same 10 specimens as the PCR-confirmed group. Percentages are calculated within each method-specific stratum; 95% confidence intervals are Wilson intervals. These figures are descriptive counts and must not be read as sensitivity estimates.*

**Supplementary Table S4.** Restricted analysis of the composite reference standard: reference-positive status limited to culture-confirmed cases and reference-negative status limited to patients with both blood and respiratory negative cultures.

| **Parameter** | **Sofia® FIA** | **LIAISON® Ag** | **Difference, pp** | **Discordant pairs in the direction of the difference, n/n (%; 95% CI); *p*** |
| --- | --- | --- | --- | --- |
| **Total cohort (n = 197): pre-resolution** | | | | |
| *2 × 2 counts against the restricted reference standard* | | | | |
| Assay-positive / reference-positive (n = 41) | 36 | 30 | — | — |
| Assay-negative / reference-positive | 5 | 11 | — | — |
| Assay-positive / reference-negative (n = 156) | 40 | 19 | — | — |
| Assay-negative / reference-negative | 116 | 137 | — | — |
| *Accuracy estimates* | | | | |
| Sensitivity, % (95% CI) | 87.8 (74.5–94.7) | 73.2 (58.1–84.3) | 14.6 | 7/8 (87.5; 47.3–99.7); *p* = 0.070 |
| Specificity, % (95% CI) | 74.4 (67.0–80.6) | 87.8 (81.8–92.1) | 13.5 | 22/23 (95.7; 78.1–99.9); *p* < 0.001 |
| PPV, % (95% CI) | 47.4 (36.5–58.4) | 61.2 (47.2–73.6) | — | — |
| NPV, % (95% CI) | 95.9 (90.7–98.2) | 92.6 (87.2–95.8) | — | — |
| LR+ (95% CI) | 3.42 (2.56–4.58) | 6.01 (3.79–9.52) | — | — |
| LR− (95% CI) | 0.16 (0.07–0.37) | 0.31 (0.18–0.51) | — | — |
| **Total cohort (n = 197): after discrepancy resolution** | | | | |
| *2 × 2 counts against the restricted reference standard* | | | | |
| Assay-positive / reference-positive (n = 41) | 34 | 30 | — | — |
| Assay-negative / reference-positive | 7 | 11 | — | — |
| Assay-positive / reference-negative (n = 156) | 36 | 19 | — | — |
| Assay-negative / reference-negative | 120 | 137 | — | — |
| *Accuracy estimates* | | | | |
| Sensitivity, % (95% CI) | 82.9 (68.7–91.5) | 73.2 (58.1–84.3) | 9.8 | 4/4 (100; 39.8–100); *p* = 0.125 |
| Specificity, % (95% CI) | 76.9 (69.7–82.8) | 87.8 (81.8–92.1) | 10.9 | 17/17 (100; 80.5–100); *p* < 0.001 |
| PPV, % (95% CI) | 48.6 (37.2–60.0) | 61.2 (47.2–73.6) | — | — |
| NPV, % (95% CI) | 94.5 (89.1–97.3) | 92.6 (87.2–95.8) | — | — |
| LR+ (95% CI) | 3.59 (2.61–4.94) | 6.01 (3.79–9.52) | — | — |
| LR− (95% CI) | 0.22 (0.11–0.44) | 0.31 (0.18–0.51) | — | — |
| **Stability cohort (n = 161): pre-resolution** | | | | |
| *2 × 2 counts against the restricted reference standard* | | | | |
| Assay-positive / reference-positive (n = 37) | 33 | 27 | — | — |
| Assay-negative / reference-positive | 4 | 10 | — | — |
| Assay-positive / reference-negative (n = 124) | 34 | 19 | — | — |
| Assay-negative / reference-negative | 90 | 105 | — | — |
| *Accuracy estimates* | | | | |
| Sensitivity, % (95% CI) | 89.2 (75.3–95.7) | 73.0 (57.0–84.6) | 16.2 | 6/6 (100; 54.1–100); *p* = 0.031 |
| Specificity, % (95% CI) | 72.6 (64.1–79.7) | 84.7 (77.3–90.0) | 12.1 | 16/17 (94.1; 71.3–99.9); *p* < 0.001 |
| PPV, % (95% CI) | 49.3 (37.7–60.9) | 58.7 (44.3–71.7) | — | — |
| NPV, % (95% CI) | 95.7 (89.6–98.3) | 91.3 (84.7–95.2) | — | — |
| LR+ (95% CI) | 3.25 (2.39–4.42) | 4.76 (3.01–7.53) | — | — |
| LR− (95% CI) | 0.15 (0.06–0.38) | 0.32 (0.19–0.54) | — | — |
| **Stability cohort (n = 161): after discrepancy resolution** | | | | |
| *2 × 2 counts against the restricted reference standard* | | | | |
| Assay-positive / reference-positive (n = 37) | 30 | 27 | — | — |
| Assay-negative / reference-positive | 7 | 10 | — | — |
| Assay-positive / reference-negative (n = 124) | 32 | 19 | — | — |
| Assay-negative / reference-negative | 92 | 105 | — | — |
| *Accuracy estimates* | | | | |
| Sensitivity, % (95% CI) | 81.1 (65.8–90.5) | 73.0 (57.0–84.6) | 8.1 | 3/3 (100; 29.2–100); *p* = 0.250 |
| Specificity, % (95% CI) | 74.2 (65.8–81.1) | 84.7 (77.3–90.0) | 10.5 | 13/13 (100; 75.3–100); *p* < 0.001 |
| PPV, % (95% CI) | 48.4 (36.4–60.6) | 58.7 (44.3–71.7) | — | — |
| NPV, % (95% CI) | 92.9 (86.1–96.5) | 91.3 (84.7–95.2) | — | — |
| LR+ (95% CI) | 3.14 (2.24–4.40) | 4.76 (3.01–7.53) | — | — |
| LR− (95% CI) | 0.25 (0.13–0.50) | 0.32 (0.19–0.54) | — | — |

*A patient was classified as reference-positive only if* S. pneumoniae *was isolated from blood culture or from an adequate-quality respiratory culture, irrespective of which other reference tests were performed. A patient was classified as reference-negative only if both a blood culture and an adequate-quality respiratory culture had been performed and neither yielded S. pneumoniae. CI, confidence interval; LR+, positive likelihood ratio; LR−, negative likelihood ratio; NPV, negative predictive value; pp, percentage points; PPV, positive predictive value.*

**Supplementary Table S5.** Effect of the discrepancy-resolution protocol on each inter-platform agreement measure, by pathogen and cohort.

| **Measure** | **Pre-resolution (95% CI)** | **After resolution (95% CI)** | **Absolute change** |
| --- | --- | --- | --- |
| ***S. pneumoniae* — Total cohort (n = 318)** | | | |
| Discordant pairs, n (%) | 47 (14.8) | 31 (9.7) | −16 |
| OPA, % | 85.2 (80.9–88.7) | 90.3 (86.5–93.0) | +5.0 |
| PPA, % | 57.7 (48.1–66.7) | 67.4 (57.3–76.1) | +9.7 |
| NPA, % | 98.6 (96.0–99.5) | 99.6 (97.5–99.9) | +1.0 |
| Cohen's kappa | 0.63 (0.53–0.72) | 0.74 (0.65–0.83) | +0.11 |
| ***S. pneumoniae* — Stability cohort (n = 255)** | | | |
| Discordant pairs, n (%) | 36 (14.1) | 25 (9.8) | −11 |
| OPA, % | 85.9 (81.1–89.6) | 90.2 (85.9–93.3) | +4.3 |
| PPA, % | 61.4 (50.9–70.9) | 69.6 (58.8–78.7) | +8.3 |
| NPA, % | 98.8 (95.7–99.7) | 99.4 (96.9–99.9) | +0.6 |
| Cohen's kappa | 0.66 (0.55–0.76) | 0.75 (0.66–0.84) | +0.09 |
| ***L. pneumophila* — Total cohort (n = 335)** | | | |
| Discordant pairs, n (%) | 22 (6.6) | 15 (4.5) | −7 |
| OPA, % | 93.4 (90.3–95.6) | 95.5 (92.7–97.3) | +2.1 |
| PPA, % | 66.7 (53.0–78.0) | 73.5 (59.7–83.8) | +6.8 |
| NPA, % | 98.2 (95.9–99.2) | 99.3 (97.5–99.8) | +1.1 |
| Cohen's kappa | 0.72 (0.60–0.83) | 0.80 (0.70–0.90) | +0.08 |
| ***L. pneumophila* — Stability cohort (n = 266)** | | | |
| Discordant pairs, n (%) | 13 (4.9) | 8 (3.0) | −5 |
| OPA, % | 95.1 (91.8–97.1) | 97.0 (94.2–98.5) | +1.9 |
| PPA, % | 60.7 (42.4–76.4) | 72.0 (52.4–85.7) | +11.3 |
| NPA, % | 99.2 (97.0–99.8) | 99.6 (97.7–99.9) | +0.4 |
| Cohen's kappa | 0.70 (0.54–0.86) | 0.80 (0.67–0.94) | +0.10 |

*CI, confidence interval; NPA, negative percent agreement; OPA, overall percent agreement; PPA, positive percent agreement. The pre-resolution column corresponds to the primary analysis and the post-resolution column to the secondary sensitivity analysis.*
